# Brugada Syndrome: Presentation and Management of the Atypical Patient in the Emergent Setting

**DOI:** 10.5811/cpcem.2020.1.44675

**Published:** 2020-04-14

**Authors:** Alexander Nguyen, Mario Flores, Vilmogil Tano

**Affiliations:** * Henry Ford Wyandotte Hospital, Department of Emergency Medicine, Wyandotte, Michigan; † Burrell College of Osteopathic Medicine at New Mexico State University, Department of Emergency Medicine, Las Cruces, New Mexico

**Keywords:** syncope, ventricular tachycardia, ventricular fibrillation, emergency department, sudden cardiac death

## Abstract

**Introduction:**

Brugada syndrome is a genetic disorder of the heart’s electrical system that increases a patient’s risk of sudden cardiac death. It is a syndrome most prevalent in Southeast Asians and is found 36 times more commonly in Asians than in Hispanics.

**Case Report:**

We report and discuss a case of a 68-year-old Hispanic male who presented with clinical and electrocardiogram abnormalities consistent with Brugada syndrome.

**Discussion:**

The patient’s age and ethnicity represents an atypical presentation of this rare syndrome and the lack of reported studies in the literature pertaining to these demographics reflect this.

**Conclusion:**

Further studies and characterizations are necessary as manifestations continue to be unearthed. As such, Brugada Syndrome should be considered in the differential diagnosis for a myriad of patient populations.

## INTRODUCTION

Brugada syndrome, an autosomal dominant disorder of the heart’s electrical system, was first described in medical literature in 1992 as a frequent cause of syncope, ventricular tachyarrhythmia, and sudden cardiac death.^2^, ^3^ An extremely rare phenomenon affecting an estimated 0.05% of people worldwide, it presents at an average age of 41 years and tends to affect men and those of Asian ancestry most frequently.^3^, ^4^ The electrocardiogram (ECG) of a patient with this syndrome classically demonstrates ST-segment elevations in leads V1 to V3 and a right bundle branch block pattern.^3^ Here we discuss a case of an elderly Hispanic patient with no significant past medical history who presented to the emergency department (ED) with first-time signs and symptoms consistent with Brugada syndrome.

## CASE REPORT

A 68-year-old Hispanic male with no significant past medical history was brought to the ED by emergency medical services (EMS) for confusion and altered mental status. EMS reported that they were called by the patient’s wife when she discovered him breathing erratically and could not rouse him. Upon arrival to the ED, the patient was obtunded and began to experience cardiac arrest with ventricular fibrillation. He was subsequently intubated and given Advanced Cardiac Life Support where cardiopulmonary resuscitation was administered and the patient was defibrillated. He went on to experience return of spontaneous circulation. An ECG obtained in the ED during this event demonstrated ST-segment elevations in V1–V3 and specific repolarization abnormalities in V1 and V2 (Image).

Additional ECG findings included the following: first-degree atrioventricular (AV) block, PR interval of 220 milliseconds (120–200 milliseconds), and QRS duration of 0.11 seconds (0.08–0.12 seconds).

The patient was placed on an amiodarone infusion and transferred to a medical facility with a cardiac catheterization laboratory. Emergent cardiac catheterization was performed due to concerns for ST-elevation myocardial infarction (STEMI), but no acute findings were discovered. Laboratory values from the initial blood draw in the ED demonstrated the following: sodium 132 milliequivalent per liter (meq/L) (normal 136–145 meq/L); potassium 3.3meq/L (normal 3.5–5.0 meq/L); magnesium 3.3 milligram per deciliter (mg/dL) (normal 1.5–2.4 mg/dL); glucose 197mg/dL; troponin <0.02 nanograms per milliliter; brain natriuretic peptide (BNP) 65; prothrombin time (PT) 13.6 seconds (normal 11–13 seconds); international normalized ratio (INR) 1.3 seconds; and thyroid stimulating hormone (TSH) 59.4 milliunits per liter (mU/L) (normal 0.5–5.0 mU/L).

Following cardiac catheterization, the patient was transferred to the intensive care unit where all vital signs remained stable and within normal limits. With members of the patient’s immediate family at bedside, information pertaining to his personal history was elicited for the first time. They reported that the patient had been in good health prior to the onset of his confusion and at no point did he complain of any discomfort or associated symptoms. Furthermore, they denied any significant past medical or surgical events in the patient’s history, including any personal or family history of heart disease or sudden cardiac death. They stated that the patient had no allergies, did not take any medications, never used tobacco or illicit drugs, and consumed alcohol occasionally during social events.

The following day, the patient was extubated with excellent response and was found to have no focal deficits on physical exam. Thyroid replacement therapy was initiated for severe hypothyroidism discovered incidentally during the aforementioned care. Although no additional adverse cardiac events would occur for the duration of his hospital stay, the patient agreed to placement of an implantable-cardioverter defibrillator (ICD) as recommended for secondary prevention of Brugada syndrome.

Further work-up of the patient during his hospital course included the following: magnetic resonance imaging of the brain, which demonstrated multiple small areas of infarct in the bilateral cerebral hemispheres and no other significant findings. Computed tomography angiography of the head and neck were found to be normal. After demonstrating an ability to ambulate 300 feet with front-wheel walker, the patient was discharged home on aspirin and statin medication.

Left heart catheterization was performed 18 days later as recommended by the patient’s electrophysiologist. The right coronary artery was found without stenosis or blockage; the left anterior descending artery was patent; and overall coronary circulation and left ventricular function were normal. These findings, coupled with the patient’s presenting symptoms and findings found on ECG in the ED, ultimately corroborated a diagnosis of Brugada syndrome with a type 1 pattern.

**CPC-EM Capsule**

What do we already know about this clinical entity?

Brugada syndrome is a rare genetic disease of the heart associated with arrhythmia and sudden cardiac death.

What makes this presentation of disease reportable?

Our Hispanic patient represents an atypical presentation of a disease most prevalent in Asians and lends credence to the need for further evaluation of this disorder.

What is the major learning point?

Brugada syndrome should be considered in the differential diagnosis for various patient populations.

How might this improve emergency medicine practice?

Broadening the differential diagnosis will aid early recognition, particularly in the elderly with unremarkable cardiac histories.

## DISCUSSION

The average age of presentation of Brugada syndrome is 41 years, and men are affected far more frequently than women (9:1 ratio).^3^ This syndrome is believed to affect 0.5 per 1000 people worldwide, with Asians affected nine times more often than Caucasians and 36 times more often than Hispanics.^1^ All patients, regardless of ethnicity, are predisposed to suffering from ventricular tachycardia, ventricular fibrillation, and sudden cardiac death.^3^ Moreover, patients are predisposed to suffering from concurrent cardiac abnormalities that include right bundle branch block, first-degree AV block, intraventricular conduction delay, and sick sinus syndrome.^4^

Several mechanisms have been implicated in the pathophysiology of Brugada syndrome. In the inherited, autosomal dominant form of the syndrome, a gene mutation alters the structure and function of sodium ion channels found in the heart. Impaired ion channels prevent the flow of sodium into the cardiac cell, which adversely affects the heart rhythm.^4^ One proposed mechanism is through mutation of the SCN5A gene, which is responsible for the production of cardiac sodium channels. The SCN5A gene is by far the most commonly affected gene found in as many as 30% of affected individuals. Conversely, other individual gene mutations are responsible for less than one percent of all presentations.^4^ In the acquired form of the syndrome, exposures from drugs and other environmental instigators alter ion levels in the blood, resulting in altered cardiac conductivity.^5^ Implicated electrolyte abnormalities include hypercalcemia, as well as hyperkalemia and hypokalemia.^5^

Diagnosis of Brugada syndrome is based on classic ECG findings that include ST-segment elevations in leads V1 to V3 and right bundle branch block patterns on ECG in conjunction with one of the following: history of ventricular tachycardia or fibrillation; family history of sudden cardiac death or a coved pattern on ECG; or agonal breathing during sleep.^3^ Our patient’s diagnosis was corroborated by way of ventricular fibrillation occurring in the ED and ST-segment elevations in leads V1 to V3 found on ECG.^3^

Unfortunately, Brugada syndrome predisposes a patient to a lifetime risk of sudden cardiac death, and there are currently no pharmacologic treatments to reduce this risk.^6^ Thus, ICD is often recommended and was the treatment modality of choice used in our patient. ICD has the greatest efficacy in averting sudden cardiac death, but the decision to use the device depends on the patient’s ability to tolerate it.^7^ If a patient cannot tolerate an ICD, pharmacologic therapy then serves as a second-line treatment modality. Pharmaceuticals can be used in the acute management of an arrhythmic storm, to prevent arrhythmia in patients with an implanted ICD requiring multiple shocks, and in cases where ICD implantation is contraindicated or not feasible – as may be the case in children or upon patient refusal.^8^

The following drugs have been found to be of great benefit: quinidine, disopyramide, quinine sulphate, beta agonists, phosphodiesterase inhibitors, bepridil, and traditional Chinese medicines such as wenxin keli and dimethyl lithospermate B.^8^ In contrast, contraindicated drugs that serve as sodium channel blockers with the ability to induce cardiac arrhythmias in Brugada syndrome include the following: ajmaline, procainamide, flecainide, propafenone and pilsicainide.^8^ Thus, it is imperative that physicians maintain Brugada syndrome atop the differential diagnosis for patients presenting with syncope, ventricular fibrillation, or STEMI, so as to avoid treating presentations with potentially underlying Brugada syndrome with a contraindicated medication.

Anticipation of Brugada syndrome in a particular patient demographic or age group should not be expected. Although it rarely presents in the geriatric and Hispanic populations and is not well studied in these individuals, it is a life threat necessitating clarity in presentation, modes of treatment, and overall mortality risk for this particular patient demographic.^9^ Further compounding this issue is the profound increase in the incidence of syncope associated with Brugada syndrome during the seventh decade of life.^9^ As the various causes of syncope are complex and multifactorial, a diagnosis of Brugada syndrome can be missed or even veiled by comorbid causes of syncope such as arrhythmias, AV blocks, and atrial fibrillation found in the elderly.^9^

There have been rare reports of SCN5A gene mutation variants that are capable of inducing Brugada syndrome under hypothyroid conditions. This is explained by thyroid hormone affecting cardiac myocyte action potential duration and repolarization currents.^10^ It remains a possibility that our patient’s clinical deterioration was incited by untreated hypothyroidism, but the association remains unclear. Future studies elucidating the relationship between hypothyroidism and Brugada syndrome are needed.

## CONCLUSION

Our patient’s age and ethnicity represent an atypical presentation of Brugada syndrome, and the lack of reported studies in the literature pertaining to this demographic reflect this. It is critically important to establish a diagnosis of Brugada syndrome in a patient lacking a personal or family history of this syndrome, as a first-time diagnosis in a single individual implicates their entire family. Furthermore, patients must be cautioned about the use of anesthetics, antihistamines, cocaine, antiarrhythmics, and psychotropic drugs as they have been found to provoke Brugada syndrome.^11^ Brugada syndrome’s abrupt symptomatology, high mortality rate, and ability to present atypically make this a challenging disorder to manage. It is for all of these reasons that Brugada syndrome will continue to serve as a significant life threat that emergency physicians will be relied upon to recognize and manage.

## Figures and Tables

**Image:**
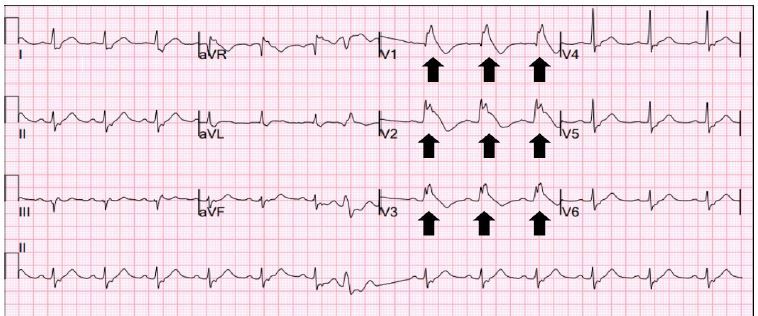
Initial patient electrocardiogram obtained in the ED. Black arrows demonstrate ST-segment elevation in a coved pattern consistent with a Type 1 Brugada pattern.
